# A study of trends and projection of life expectancy and its association with socio-demographic index: Results from GBD study 2023

**DOI:** 10.1371/journal.pone.0347865

**Published:** 2026-06-03

**Authors:** Reza Taherian, Maryam Karimi Ghahfarokhi, Farid Zayeri

**Affiliations:** 1 Department of Biostatistics, School of Allied Medical Sciences, Shahid Beheshti University of Medical Sciences, Tehran, Iran; 2 Proteomics Research Center, Department of Biostatistics, Faculty of Allied Medical Sciences, Shahid Beheshti University of Medical Sciences, Tehran, Iran; Facultad Latinoamericana de Ciencias Sociales Mexico, MEXICO

## Abstract

**Background:**

Life expectancy (LE) serves as a central indicator of population health, reflecting the combined effects of social, economic, and demographic factors. Despite global improvements, substantial disparities persist across regions and development levels. This study aims to analyze temporal and spatial trends in life expectancy at birth (LEB) and assess its relationship with the Socio-Demographic Index (SDI) from 1960 to 2023, with projections to 2050.

**Methods:**

LE data for 190 countries from 1960 to 2023 were obtained from Our World in Data, while the SDI, reflecting overall social and economic development, was derived from GBD data. Joinpoint regression models were used to estimate annual and average annual percentage changes (APC, AAPC) across countries, regions, and SDI groups. Correlation analyses assessed the strength of association between LEB and SDI. Future trends were projected using a Bayesian Age-Period-Cohort (BAPC) model, generating estimates up to 2050 with 95% credible intervals.

**Results:**

Global LE increased steadily from 1960 to 2023, with an overall AAPC of 0.58%. Despite this progress, large regional and socio-demographic gaps persist. Lower-SDI and low-income regions exhibited faster relative growth but greater fluctuations, whereas higher-SDI regions demonstrated slower yet stable improvement. The correlation between LEB and SDI remained strong throughout the study period (r = 0.82–0.90). Projections from the BAPC model indicate continued increases in LE, from 73.6 years in 2025 to 82.8 years in 2050, although uncertainty widens toward mid-century, reflecting potential divergence in global health trajectories.

**Conclusion:**

LE has improved globally but remains uneven across socio-demographic contexts. Strengthening social and economic systems, expanding equitable healthcare access, and integrating SDI-based evidence into policy frameworks are essential to sustain progress toward health equity and longevity through 2050 and beyond.

## Introduction

Life expectancy (LE) is one of the most important indicators for assessing the overall health status and well-being of populations. It reflects the average number of additional years a person is expected to live from a given age onward, assuming that current age-specific mortality rates remain constant. Among various measures of population health, life expectancy at birth (LEB) is the most widely used metric, defined as the average number of years a newborn is expected to live under prevailing mortality conditions [1]. This indicator not only provides valuable insights into public health outcomes but also shows a strong association with the Socio-Demographic Index (SDI) [2,3].

According to the latest global health statistics, the average LEB for the world’s population reached 73.3 years in 2024, representing an improvement of more than 8.4 years since 1995 [4,5]. Projections suggest that if current survival trends continue, the global average LE may reach 77.4 years by 2050 [4–6]. Despite these significant achievements, large disparities persist across regions and within countries. On average, LEB in low- and lower-middle-income countries remains about 7.4 years below the global average, largely due to higher child and maternal mortality rates, poverty, malnutrition, limited access to healthcare services, and lower educational attainment [7,8]. For instance, according to the latest available data (2023) from the World Bank’s World Development Indicators, Monaco reported one of the highest life expectancies globally, exceeding 86 years, followed by San Marino with similarly high values. In contrast, several Sub-Saharan African countries, including Nigeria and Chad, reported life expectancy levels below 56 years during the same period [4,9]. This persistent gap of nearly 30 years highlights the strong influence of socioeconomic development, education, healthcare accessibility, and demographic dynamics on population health outcomes [10].

To analyze trends in LEB, previous studies have commonly employed demographic and statistical techniques such as life table analysis and specific longitudinal trend modeling approaches, as well as indicators like the healthy life expectancy index (HALE) to examine patterns in longevity and population health over time [11–13]. Although LE generally shows an increasing trend, this improvement is strongly influenced by various factors, including access to healthcare services, economic development, educational attainment, public health policies, and social equity [14]. For each of these factors, specific indicators are available; for example, the Universal Health Coverage Index (UHC Index) for healthcare access, gross domestic product per capita (GDP per capita) for economic development, mean years of schooling for education, and the Human Development Index (HDI) for overall socio-economic development. Among these indicators, the SDI holds particular importance. Developed by the Institute for Health Metrics and Evaluation (IHME), SDI combines three key components-income per capita, mean years of schooling, and total fertility rate-and is widely applied in Global Burden of Disease (GBD) studies to compare and analyze the socio-demographic development levels of countries and regions [15]. The advantage of SDI over many other indices is that it simultaneously captures economic, educational, and demographic dimensions while maintaining a strong association with important health indicators such as the burden of disease (DALY) and LE.

Although numerous studies have examined the global patterns of LEB and its association with socio-demographic factors, important gaps still persist. For example, Zheng et al. in 2024 analyzed life and healthy life disparity across countries in relation to social indices, yet did not explicitly explore longitudinal trends linking LEB and SDI [16]. While projection efforts such as Mathers et al. in 2006 provide forecasts for global mortality and LE, they often lack integration with socio-demographic indices like SDI in a unified framework [17]. The GBD 2015 study analyses incorporate SDI in cause-of-death modeling and expected mortality patterns (e.g., “observed vs expected” mortality by SDI) [18], but comprehensive studies spanning multi-decade trends (e.g., 1960 to present) and explicitly assessing the strength and dynamics of LEB-SDI relationships remain scarce. Moreover, while future projections for LE (e.g., Vollset et al. in 2024) account for demographic and epidemiological changes [19], few studies incorporate multiple socio-demographic scenarios or examine regional heterogeneity systematically. Furthermore, much of the work on disease burden (e.g., Kyu et al., DALYs by SDI) [20] and HALE projections (e.g., for 202 countries to 2030) [21] provides insight into health outcomes, but not explicitly the lagged or bidirectional relationships with LEB over long timescales. Finally, advanced forecasting techniques (e.g., Shang’s multilevel functional data methods) [22] have often been applied to high-income contexts, limiting their generalizability to a global, socio-demographically diverse setting.

To address the above-mentioned gaps, the present study aims to analyze global trends in life expectancy at birth (LEB) from 1960 to 2023 and examine its association with the Socio-Demographic Index (SDI) across countries and regions. Using comprehensive global datasets, we apply Joinpoint regression to assess temporal trends, Pearson correlation analysis to evaluate the relationship between LEB and SDI, and the Bayesian Age–Period–Cohort (BAPC) model to project life expectancy at selected ages (birth, 10, 25, 45, 60, and 80 years) through 2050. This integrated analytical framework allows us to assess long-term patterns, regional disparities, and age-specific future trajectories of life expectancy.

The remainder of this paper is organized as follows. The Methods section describes the data sources, variable definitions, and statistical approaches used in the analysis. The Results section presents global and regional trends in life expectancy, evaluates its association with SDI, and provides age-specific projections through 2050. The Discussion section interprets the findings in the context of previous research and outlines policy implications as well as methodological strengths and limitations. Finally, the Conclusion summarizes the key findings and their implications for global health planning.

## Methods

### Data source

LEB data were obtained from publicly available international databases, covering the period 1960–2023. Specifically, life expectancy estimates were obtained from Our World in Data, which compiles harmonized data primarily from the World Bank’s World Development Indicators, the United Nations Population Division, and the World Health Organization [23]. The Socio-Demographic Index (SDI) was obtained directly from the Global Burden of Disease (GBD) study conducted by the Institute for Health Metrics and Evaluation (IHME, https://vizhub.healthdata.org/gbd-results/), which is the official source for SDI classification. The GBD database provides complementary data from 204 countries and territories spanning 1960–2023, categorized by cause of death, risk factors, age group, sex, and the SDI. These sources are widely recognized and extensively used in global health and demographic research. All datasets used in this study were accessed for research purposes on 12 October 2025, and the authors did not have access to any information that could identify individual participants at any stage of data collection or analysis.

A total of 190 countries with available LEB and SDI data, spanning all five continents, were included in the analysis. Using these datasets, we examined the relationship between SDI and LEB over time and across regions to assess how variations in sociodemographic development influence population health outcomes globally.

Analyses were restricted to countries and years with complete data from GBD 2023, and no additional data cleaning or imputation was performed.

After compiling the datasets described above, we defined the main outcome and supporting variables used for analysis as follows.

### Main outcome and other variables under study

LE is commonly estimated using two primary approaches: cohort-based and period-based methods. Cohort LE tracks a group of individuals born in the same year throughout their lives, providing a longitudinal perspective on mortality. In contrast, period LE relies on current mortality rates to estimate the expected lifespan of a hypothetical cohort, making it a more widely used approach due to the limited availability of complete cohort data. Estimating LE for large regions or global populations, however, poses significant challenges, as neither cohort nor period data alone can always provide accurate results. In such cases, researchers often combine both types of data to construct life tables and derive more reliable estimates [1,24,25].

To provide a comprehensive analysis and contextualize the LEB-SDI relationship, the study incorporated several other key variables for stratification and comparison. These included:

**Geographic and Demographic Classifications:** Analyses were stratified by continent (Africa, Americas, Asia, Europe, Oceania), the eight predefined GBD regions (Africa Eastern and Southern: AFE, Africa Western and Central: AFW, Central Europe and the Baltics: CEB, East Asia and Pacific: EAP, Latin America and Caribbean: LCN, Middle East and North Africa: MEA, North America: NAC, and South Asia: SAS), and gender (male, female) to examine spatial and demographic disparities.

**Economic Development:** This index is measured on a scale from 0 to 100 and reflects a country’s overall social and economic development by combining indicators like income per capita, average educational attainment, and fertility rates. Based on their SDI values, countries are classified into five categories: high, high-middle, middle, low-middle, and low, which helps in understanding and comparing health outcomes relative to socioeconomic status.

**Age Groups:** For the projection modeling using the BAPC framework, LEB was analyzed and forecast for specific age groups (at birth, 10, 25, 45, 65, and 80 years) to understand age-specific trends in longevity.

### Statistical analysis

We employed Joinpoint regression analysis to evaluate temporal trends and to identify potential points of significant change in slope. Joinpoint regression is a statistical method used to identify significant changes in temporal trends by fitting segmented linear models to the data. It is commonly applied in epidemiological and demographic studies to detect changes in indicators such as mortality and life expectancy over time. The model fits a series of linear segments to the data, with each segment connected at estimated joinpoints. The number and location of joinpoints were determined using a permutation test with an overall significance level of 0.05. In the present study, this framework was applied to assess long-term patterns in LEB and the SDI. For each segment, the Annual Percent Change (APC) with its 95% confidence interval was estimated to quantify the direction and magnitude of change, and when multiple joinpoints were identified, the Average Annual Percent Change (AAPC) was calculated to provide a summary measure across the study period [26]. Data analyses were performed using Joinpoint Regression software (version 5.4.0.0).

We also applied the Bayesian Age-Period-Cohort (BAPC) model to estimate LEB from 1960 to 2023 and predict this index for the period of 2024–2050 [27]. These models assume that LEB for a given age group and time period can be modeled based on age, period, and cohort effects, which may follow linear or random-walk trends [28,29], providing a flexible framework to capture temporal patterns over the life course. In this framework, age effects reflect changes across an individual’s lifespan, period effects capture changes affecting all age groups simultaneously, and cohort effects describe differences among successive birth cohorts. The BAPC-based projections assume continuity of historical age-period-cohort patterns and do not explicitly account for potential future large-scale social, economic, or health shocks (e.g., pandemics or conflicts). Accordingly, the projected estimates are intended to be interpreted as plausible scenario-based extrapolations of past trends rather than precise predictions of future life expectancy.

Forecast uncertainty was quantified using posterior distributions, with 95% credible intervals (CrIs) derived to reflect the range of plausible future values. To account for population heterogeneity, projections were stratified by age groups (at birth, 10, 25, 45, 65, and 80 years), providing age-specific insights into future trends in LEB.

Model estimation and prediction were carried out through the Integrated Nested Laplace Approximation (INLA), a method that enables efficient and precise Bayesian inference for latent Gaussian models. All analyses were implemented using the INLA package in R (version 4.5.1).

Finally, to assess the relationship between the SDI and LEB across regions and over time, Pearson’s correlation coefficient was calculated. This method allowed us to quantify the strength and direction of the linear association between SDI and LEB globally.

### Ethical approval

The study received approval from the Ethics Committee of Shahid Beheshti University of Medical Sciences under approval number IR.SBMU.RETECH.REC.1404.565.

## Results

To investigate the trends of LEB across global regions, we first described the pattern of changes from 1960 to 2023. These descriptive results are summarized for the total populations of each region to highlight long-term shifts. Table 1 presents LEB at the beginning and end of the study period, along with the AAPC by gender, continent, global region, income group, and SDI level. Detailed tables for each subgroup are provided in the S1-S5 Tables.

**Table 1:** Life expectancy at birth in 1960 and 2023, and average annual percent change (AAPC) from 1960 to 2023, by different characteristics.

	Life expectancy at birth		1960-2023 AAPC (95% CI)
	1960	2023	
**Gender**			
Female	53.03	75.84	0.58 (0.57,0.59)
Male	49.03	70.95	0.60 (0.58,0.61)
Both	50.94	73.33	0.59 (0.57,0.60)
**Continent/Region**			
Africa	41.40	63.84	0.68 (0.66,0.70)
Africa Eastern and Southern	44.17	65.15	0.60 (0.59,0.62)
Africa, Western and Central	37.78	58.86	0.70 (0.69,0.71)
Americas	60.79	77.29	0.38 (0.37,0.39)
Latin America and the Caribbean	55.08	75.64	0.51 (0.50,0.52)
North America	69.89	78.73	0.18 (0.18,0.19)
Asia	41.79	74.57	0.92 (0.90,0.95)
East Asia and the Pacific	41.71	76.66	0.98 (0.97,0.99)
Middle East and North Africa	43.52	72.08	0.80 (0.77,0.82)
South Asia	45.58	72.32	0.75 (0.70,0.78)
Europe	68.71	79.06	0.22 (0.21,0.23)
Central Europe and the Baltics	67.94	77.90	0.22 (0.21,0.24)
Oceania	65.04	79.15	0.31 (0.30,0.31)
**Income group**			
Low income	41.62	64.52	0.68 (0.67,0.69)
Low-middle income	45.83	69.60	0.67 (0.64,0.69)
Middle income	43.57	72.74	0.83 (0.81,0.84)
High-middle income	41.96	76.20	0.96 (0.95,0.98)
High income	68.34	80.15	0.25 (0.25,0.26)
**Socio-demographic index**			
Low SDI	48.72	61.78	0.39 (0.38,0.40)
Low-middle SDI	67.21	66.55	0.01 (−0.00,0.02)
Middle SDI	71.91	70.83	−0.00 (−0.03,0.01)
High-middle SDI	71.59	74.80	0.07 (0.06,0.09)
High SDI	74.67	80.34	0.17 (0.15,0.18)

**Fig 1:**
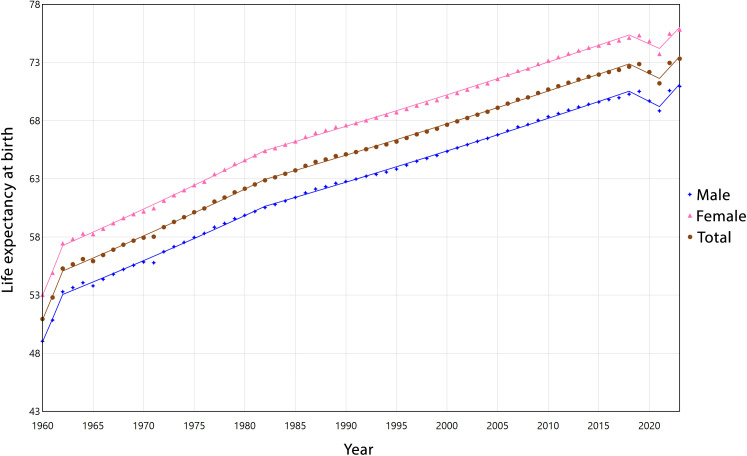
Estimated trends in life expectancy at birth from 1960 to 2023 by Gender.

Overall, LEB increased steadily from 1960 to 2018, with higher growth in the early 1960s (APC = 4.04% for the total population). A temporary decline occurred during 2018–2021 (APC = −0.58%), followed by renewed growth in 2021–2023 (APC = 1.32%). Both females and males showed similar trends over time, with slightly higher long-term relative growth in males (overall AAPCs of 0.60% vs 0.58%). Despite this, females consistently had higher LE than males throughout the study period. Fig 1 illustrates the trends in life expectancy at birth (LEB) by gender from 1960 to 2023, with detailed period-specific results provided in S1 Table.

**Fig 2:**
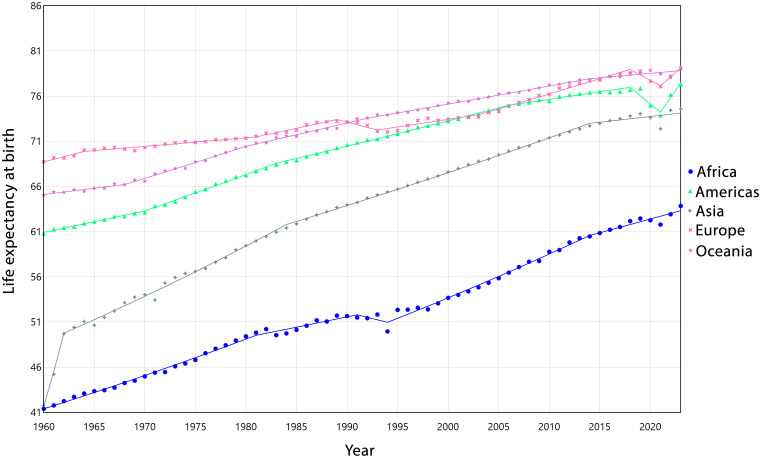
Estimated trends of life expectancy in five continents from 1960 to 2023.

LEB improved across all continents between 1960 and 2023, although the pace of increase varied. Africa experienced the highest long-term growth (AAPC = 0.68%, 95%CI: 0.66,0.70), followed by Asia (0.92%, 95%CI: 0.90,0.95), the Americas (0.38%, 95%CI: 0.37,0.39), and Oceania (0.31%, 95% CI: 0.30, 0.31). In contrast, Europe showed more modest gains (AAPC = 0.22%, 95% CI: 0.21,0.23). Fig 2 illustrates the trends in LEB across the five continents from 1960 to 2023, with detailed segmented APC analyses for each continent provided in S2 Table.

**Fig 3:**
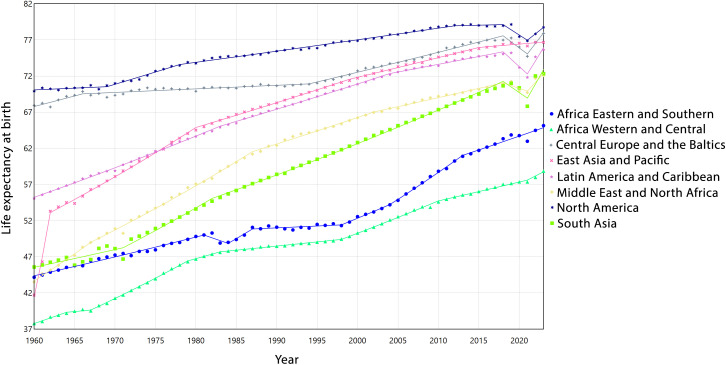
Estimated trends of life expectancy in eight regions from 1960 to 2023.

LEB improved across all regions between 1960 and 2023, although the pace of increase varied. Steep increases occurred during different periods across regions: EAP experienced an exceptionally high rise in the early 1960s (1960−1962, APC = 13.23, 95% CI: 12.87,13.83), MEA and AFW showed strong growth during the 1960s-1980s, and SAS exhibited continuous growth until the late 2010s, interrupted briefly in 2018−2021. Some short-term declines were observed, such as in CEB (2018−2021, APC = −1.09, 95%CI: −1.31,-0.66), LCN (2018−2021, APC = −1.37, 95%CI: −1.55,-1.12), NAC (2018−2021, APC = −0.92, 95%CI: −1.12,-0.62), and SAS (2018−2021, APC = −1.10, 95%CI: −1.71,0.01), indicating temporary slowdowns in LEB growth. Long-term AAPC values differed among regions. AFW (0.70, 95%CI: 0.69,0.71), MEA (0.80, 95%CI: 0.77,0.82), and SAS (0.75, 95%CI: 0.70,0.78) experienced the highest overall growth, whereas NAC (0.18, 95%CI: 0.18,0.19) and CEB (0.22, 95%CI: 0.21,0.24) had the lowest, despite occasional short-term accelerations. Periods of decline were generally followed by recovery with relatively high APCs, indicating resilience and a return to the upward trend. Fig 3 illustrates the trends in life expectancy at birth (LEB) across the eight global regions from 1960 to 2023, with detailed segmented APC analyses provided in S3 Table.

**Fig 4:**
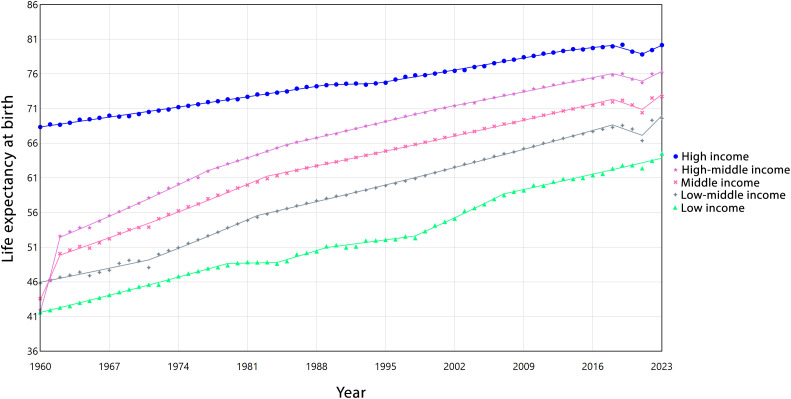
Estimated trends of life expectancy from 1960 to 2023 by income.

Fig 4 illustrates the trends in LEB across different income groups, with detailed numerical values provided in S4 Table.LEB increased in all groups, though the pace and stability of growth differed. Low and lower-middle-income countries showed substantial long-term gains (AAPC = 0.68%, 95%CI: 0.67,0.69; and 0.67%, 95%CI: 0.64,0.69, respectively), despite temporary declines around 2018–2021. Middle-income countries also experienced notable improvements (AAPC = 0.83%, 95%CI: 0.81,0.84), while high-middle-income countries showed the fastest overall growth (AAPC = 0.96%, 95%CI: 0.95,0.98). In contrast, high-income countries had slower but more stable gains (AAPC = 0.25%, 95%CI: 0.25,0.26). Overall, a distinct income-related gradient was observed, with lower-income regions showing rapid but fluctuating progress, whereas higher-income regions demonstrated gradual and sustained improvements in LE.

**Fig 5:**
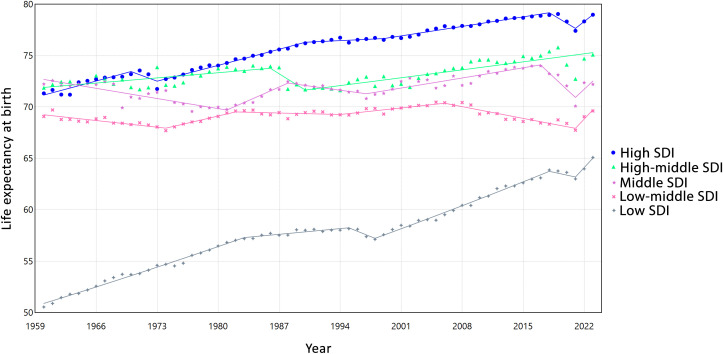
Estimated trends in life expectancy at birth from 1960 to 2023 by Socio-Demographic Index.

As the main objective of this study, we also modeled the pattern of change in LEB in different levels of SDI. Fig 5 illustrates the estimated trends in LEB across different Socio-SDI categories, with detailed numerical values provided in S5 Table. LEB increased in all SDI groups, although the magnitude and stability of growth varied. The Low SDI countries showed steady improvement (AAPC = 0.39%, 95%CI: 0.38,0.40), with short-term declines during 1995–1998 and 2018–2021, followed by recovery after 2021. The Low-middle SDI countries exhibited nearly stagnant growth (AAPC = 0.01%, 95%CI: −0.00,0.02), with modest gains after 2021. For the Middle SDI group, trends remained stable (AAPC≈0.00%, 95%CI: −0.03,0.01), showing alternating mild increases and declines. In the High-middle SDI group, LEB rose gradually (AAPC = 0.07%, 95%CI: 0.06,0.09) following a brief dip during the late 1980s. Finally, the High SDI Countries experienced moderate but consistent gains (AAPC = 0.17%, 95% CI: 0.15, 0.18), interrupted by short-term declines around 1970–1973 and 2018–2021. Overall, Lower-SDI countries achieved faster relative improvements, whereas higher-SDI countries showed slower but steadier progress.

**Fig 6:**
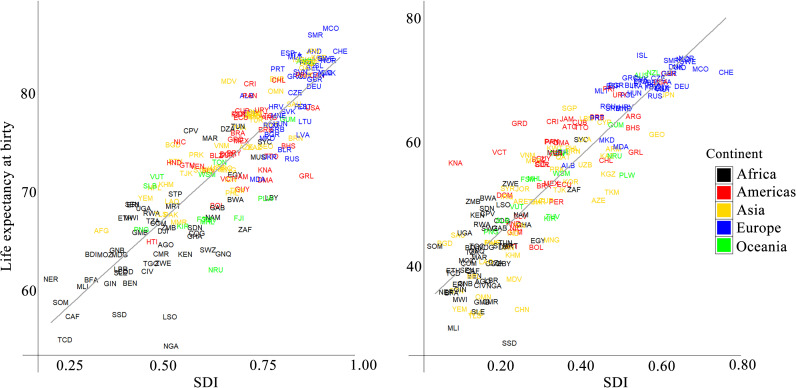
Relationship between Socio-Demographic Index (SDI) and life expectancy at birth (LEB) across countries (left:1960, right: 2023)‌‌.

In the next step of data analysis, to describe the association between SDI and LEB during the study period, we computed Pearson’s correlation coefficient between these two indices for the years 1960–2023. As shown in S6 Table, the correlation coefficients between variables remained consistently high throughout the study period, ranging from 0.82 to 0.90. This indicates a strong and stable positive association over time. Correlations were slightly higher during the 1960s (around 0.90) and tended to decline marginally after 2000, reaching approximately 0.85 in recent years. Despite minor year-to-year fluctuations, the overall pattern suggests a persistent and robust relationship across the entire 1960–2023 period. Fig 6 shows the country-level relationship between the SDI and LEB in 1960 and 2023.

**Fig 7:**
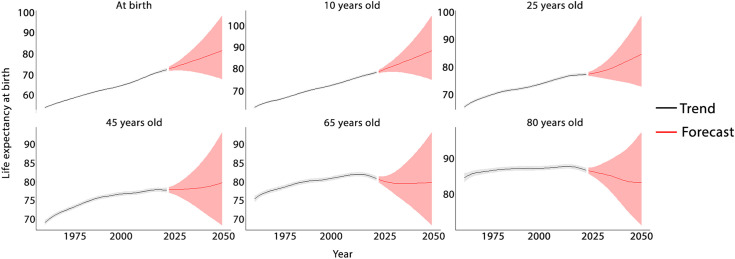
Projected life expectancy at selected ages (birth, 10, 25, 45, 60, and 80 years) from 1960 to 2050.

In the final step of data analysis, we utilized the BAPC model to project the LEB values for different ages from 2024 to 2050. Fig 7 illustrates projected life expectancy at selected ages (birth, 10, 25, 45, 60, and 80 years) from 1960 to 2050, based on the BAPC model. The projections show notable variation in LEB across ages and future time points. In 2025, LEB at birth is estimated at 73.6 years (95% CrIs: 71.95, 75.30), rising to 75.3 in 2030 (72.37, 78.49), 79.0 (75.16, 86.47) in 2040, and 82.8 (70.97, 96.55) in 2050. Similar increasing trends are observed at ages 10, 25, 45, 60, and 80. Full estimates with 95% credible intervals from 1960 to 2050 are available in S1 File.

## Discussion

This analysis examines global and regional trends in LEB from 1960 to 2023, revealing substantial improvements across all world regions, although the pace of progress has varied significantly. The global average LEB increased from approximately 50.9 years in 1960 to 73.3 years in 2023, largely driven by advancements in public health, expanded access to healthcare, reductions in infant and child mortality, and improved control of infectious diseases. Despite these overall gains, persistent disparities remain across regions and socio-demographic groups. Higher SDI levels are strongly associated with longer life expectancy, yet the magnitude of this relationship differs [30]. Utilizing comprehensive data from the GBD study, this analysis not only evaluates historical patterns but also provides forecasts to 2050, offering valuable insights for global and regional health policy development.

Regarding the obtained results about increasing global and regional trends of LEB in our study, other research in different parts of the world also shows that LEB patterns have a substantial progress worldwide, though the magnitude and stability of these improvements differ markedly across regions [31–33]. According to our findings, regions such as AFW and SAS experienced consistent upward trends, reflected in relatively high AAPC values (0.70% and 0.75%, respectively). In contrast, CEB, LCN, and NAC exhibited periods of stagnation or even decline, most notably during 2018−2021, likely influenced by health crises, including the COVID-19 pandemic [34]. EAP showed rapid initial gains in the 1960s, followed by a gradual deceleration, highlighting the diminishing marginal improvements in already high-SDI settings [35]. These heterogeneous trajectories underscore that while global LE has risen overall, regional disparities remain evident, shaped by diverse demographic, epidemiological, and socio-economic contexts.

Examining LE by gender reveals similar overall trends for males and females, with important nuances. Both genders experienced rapid gains in the early 1960s and steady improvement until 2018. The decline during 2018–2021 was more pronounced among males, likely reflecting higher vulnerability to health crises such as the COVID-19 pandemic [36]. Females consistently live longer than males, but the slightly faster long-term gains in males (AAPC: 0.60% vs. 0.58%) suggest a gradual narrowing of the gender gap. This convergence may result from reductions in male mortality from infectious diseases and risky behaviors [37]. The partial rebound in 2021–2023 highlights short-term recovery after the pandemic, though sustained structural improvements remain essential to maintain these gains.

Analyzing LE trends across different SDI categories reveals distinct temporal patterns shaped by socio-economic development. Low SDI countries experienced steady but modest gains until the late 1970s, followed by periods of stagnation and even slight declines, likely reflecting vulnerability to infectious diseases and limited healthcare infrastructure. Recovery phases in the early and mid-2000s highlight the impact of targeted health interventions and international support [38,39]. In contrast, middle and high-middle SDI countries showed generally positive trends over the long term, albeit interrupted by short-term declines during periods of economic or health crises. High SDI countries demonstrated the most stable gains, although even they were affected by recent shocks such as the COVID-19 pandemic [16,40], which caused temporary declines in life expectancy. Overall, these heterogeneous trajectories indicate that while global LE has increased, the magnitude and resilience of improvements strongly depend on the socio-economic context. Countries with lower SDI face persistent challenges in sustaining long-term gains, whereas higher SDI settings benefit from structural health and social advantages that buffer against temporary setbacks. This contrast may also reflect underlying developmental dynamics. In lower-SDI countries, improvements often occur from a relatively low baseline; therefore, reductions in child mortality, expansion of primary healthcare, vaccination programs, and control of infectious diseases can generate comparatively rapid gains in life expectancy. Such progress, however, may remain vulnerable to economic instability, health system fragility, or external shocks, resulting in greater temporal fluctuations. In contrast, high-SDI countries, where life expectancy has already reached advanced levels, tend to experience slower but more stable increases. At these stages, further gains depend largely on incremental improvements in chronic disease management, technological innovation, and elderly care, suggesting a potential ceiling effect in longevity expansion. Together, these catch-up and ceiling dynamics provide a plausible explanation for the observed differences in growth patterns across SDI strata and highlight the need for targeted, SDI-specific interventions tailored to each country’s socio-demographic context to sustain gains, reduce vulnerability, and guide long-term health policy.

Correlation analyses between SDI and LE revealed a consistently strong positive association across the study period, supporting the Joinpoint regression findings. Taken together, these complementary results indicate that SDI not only drives higher LE but also enhances the stability and resilience of health outcomes, protecting populations against global disruptions such as economic recessions or pandemics.

The BAPC model projections reveal notable variations in LE gains across age groups between 2025 and 2050. While LEB shows a substantial increase of about 12.5% (from 73.6 to 82.8 years), the pace of improvement diminishes with advancing age. Younger cohorts (ages 0−25) experience the largest projected gains, reflecting ongoing improvements in child and young adult survival, consistent with the global reductions in premature mortality reported by the GBD 2021 study. In contrast, older age groups (45−80 years) show smaller or plateauing changes, suggesting that advances in longevity at later ages may be reaching a saturation point due to chronic disease burdens and biological limits. These disparities across ages mirror the findings of Aburto et al. in 2021, which highlighted the uneven recovery of LE after the COVID-19 pandemic [40], and Zheng and Canudas-Romo in 2024, who emphasized persistent global inequalities in health and longevity [16]. Overall, the projections suggest continued improvement in life expectancy, driven primarily by younger age survival gains, but underscore the need for policies targeting healthy aging and reducing health disparities among older populations.

In this study, Joinpoint regression provided a robust approach to analyze historical trends in LEB and the SDI, allowing the identification of significant change points that might be overlooked by simple linear or polynomial models [41]. For forecasting LEB, the BAPC model with INLA offered flexible long-term predictions with credible intervals, addressing some limitations of classical APC or Lee-Carter models that often assume linear trends and provide limited uncertainty quantification [42–44]. Compared with previous studies, this combination allows both precise historical trend detection and reliable future projections. However, limitations include the usual assumptions in Joinpoint regarding linearity within segments and in BAPC regarding the structure of age, period, and cohort effects, which may not capture all complex population dynamics. Despite these constraints, the integrated approach strengthens the study by providing a more comprehensive understanding of trends and potential future patterns in population health.

## Conclusion

This study provides a comprehensive assessment of global and regional trends in LEB from 1960 to 2023 and examines its relationship with the SDI, with projections extending to 2050. Our findings demonstrate a sustained global increase in life expectancy over the past six decades, although progress has been uneven across regions and socio-demographic groups. While lower-SDI countries experienced relatively faster proportional gains, these improvements were often accompanied by greater volatility and vulnerability to external shocks. In contrast, higher-SDI countries showed more stable but slower incremental growth. A consistently strong positive association between SDI and LEB across the study period underscores the fundamental role of socioeconomic development, education, fertility patterns, and structural health determinants in shaping population longevity. The temporary declines observed during 2018–2021 highlight the sensitivity of life expectancy to global crises, emphasizing the need for resilient health systems and social protection mechanisms. Projections from the BAPC model suggest a continued increase of 9.2 years in LE in 2050-though uncertainty widens toward mid-century. These findings underscore the need for targeted strategies to reduce regional gaps in life expectancy, including sustained investment in primary healthcare, maternal and child health, education, and health system resilience, particularly in lower-SDI settings, to promote more equitable and sustainable longevity gains worldwide.

## Supporting information

S1 Table Results of the Joinpoint regression models for trend analysis of life expectancy at birth by gender from 1960 to 2023.
(DOCX)

S2 Table Results of the Joinpoint regression models for trend analysis of life expectancy at birth by continent from 1960 to 2023.
(DOCX)

S3 Table Results of the Joinpoint regression models for trend analysis of life expectancy at birth by region from 1960 to 2023.
(DOCX)

S4 Table Results of the Joinpoint regression models for trend analysis of life expectancy at birth by SDI from 1960 to 2023.
(DOCX)

S5 Table Results of the Joinpoint regression models for trend analysis of life expectancy at birth by income group from 1960 to 2023.
(DOCX)

S6 Table Year-wise correlation between the Socio-Demographic Index and life expectancy at birth from 1960 to 2023.
(DOCX)

S1 File Predicted life expectancy at birth, 10, 25, 45, 60, and 80 years with 95% credible intervals, 1960–2050.
(CSV)
