# Supplementary material for: A study of trends and projection of life expectancy and its association with socio-demographic index: Results from GBD study 2023
Source: PLoS One. 2026 Jun 3;21(6):e0347865. doi: 10.1371/journal.pone.0347865 (PMC13232855; doi:10.1371/journal.pone.0347865)
Supplement: S1 Table — Results of the Joinpoint regression models for trend analysis of life expectancy at birth by gender from 1960 to 2023. (DOCX) [file pone.0347865.s001.docx]

**S1 Table. Results of the Joinpoint regression models for trend analysis of life expectancy at birth by gender from 1960 to 2023.**

| Gender | Trend | Period | APC (95% CI) |
| --- | --- | --- | --- |
| Female | Trend 1 | 1960-1962 | 3.10* (3.72, 4.29) |
|  | Trend 2 | 1962-1982 | 0.67* (0.65, 0.69) |
|  | Trend 3 | 1982-2018 | 0.39* (0.39, 0.40) |
|  | Trend 4 | 2018-2021 | -0.52* (-0.67, -0.23) |
|  | Trend 5 | 2021-2023 | 1.22* (0.86, 1.54) |
|  | **AAPC** | **1960-2023** | **0.58* (0.57, 0.59)** |
| Male | Trend 1 | 1960-1962 | 4.09* (3.77, 4.49) |
|  | Trend 2 | 1962-1982 | 0.67* (0.65, 0.70) |
|  | Trend 3 | 1982-2018 | 0.42* (0.41, 0.43) |
|  | Trend 4 | 2018-2021 | -0.63* (-0.82, -0.29) |
|  | Trend 5 | 2021-2023 | 1.39 * (0.92, 1.79) |
|  | **AAPC** | **1960-2023** | **0.60* (0.58, 0.61)** |
| Total | Trend 1 | 1960-1962 | 4.04* (3.76, 4.38) |
|  | Trend 2 | 1962-1982 | 0.67* (0.65, 0.69) |
|  | Trend 3 | 1982-2018 | 0.41* (0.40, 0.42) |
|  | Trend 4 | 2018-2021 | -0.58* (-0.74, -0.27) |
|  | Trend 5 | 2021-2023 | 1.32* (0.92, 1.67) |
|  | **AAPC** | **1960-2023** | **0.59* (0.57, 0.60)** |
| APC: Annual Percent Change  AAPC: Average Annual Percent Change | | | |
