# Supplementary material for: A study of trends and projection of life expectancy and its association with socio-demographic index: Results from GBD study 2023
Source: PLoS One. 2026 Jun 3;21(6):e0347865. doi: 10.1371/journal.pone.0347865 (PMC13232855; doi:10.1371/journal.pone.0347865)
Supplement: S2 Table — Results of the Joinpoint regression models for trend analysis of life expectancy at birth by continent from 1960 to 2023. (DOCX) [file pone.0347865.s002.docx]

**S2 Table. Results of the Joinpoint regression models for trend analysis of life expectancy at birth by continent from 1960 to 2023.**

| Continent | Trend | Period | APC |
| --- | --- | --- | --- |
| Africa | Trend 1 | 1960-1981 | 0.86* (0.80, 0.95) |
|  | Trend 2 | 1981-1991 | 0.45* (0.18, 0.82) |
|  | Trend 3 | 1991-1994 | -0.54 (-0.85, 0.87) |
|  | Trend 4 | 1994-2014 | 0.87* (0.76, 1.03) |
|  | Trend 5 | 2014-2023 | 0.49* (0.15, 0.70) |
|  | **AAPC** | **1960-2023** | **0.68* (0.66, 0.70)** |
| America | Trend 1 | 1960-1970 | 0.38* (0.32, 0.43) |
|  | Trend 2 | 1970-1983 | 0.62* (0.58, 0.69) |
|  | Trend 3 | 1983-2006 | 0.39* (0.37, 0.41) |
|  | Trend 4 | 2006-2018 | 0.22* (0.17, 0.26) |
|  | Trend 5 | 2018-2021 | -1.21* (-1.36, -1.01) |
|  | Trend 6 | 2021-2023 | 2.17* (1.83, 2.46) |
|  | **AAPC** | **1960-2023** | **0.38* (0.37, 0.39)** |
| Asia | Trend 1 | 1960-1962 | 9.31* (8.76, 10.23) |
|  | Trend 2 | 1962-1984 | 0.99* (0.95, 1.03) |
|  | Trend 3 | 1984-2014 | 0.56* (0.53, 0.58) |
|  | Trend 4 | 2014-2023 | 0.17 (-0.04, 0.30) |
|  | **AAPC** | **1960-2023** | **0.92 (0.90, 0.95)** |
| Europe | Trend 1 | 1960-1964 | 0.42* (0.20, 0.79) |
|  | Trend 2 | 1964-1981 | 0.13 (0.01, 0.16) |
|  | Trend 3 | 1981-1989 | 0.34* (0.24, 0.58) |
|  | Trend 4 | 1989-1993 | -0.40* (-0.66, -0.15) |
|  | Trend 5 | 1993-2003 | 0.23* (0.13, 0.31) |
|  | Trend 6 | 2003-2018 | 0.44* (0.41, 0.50) |
|  | Trend 7 | 2018-2021 | -0.77* (-0.96, -0.44) |
|  | Trend 8 | 2021-2023 | 1.25* (0.88, 1.61) |
|  | **AAPC** | **1960-2023** | **0.22* (0.21, 0.23)** |
| Oceania | Trend 1 | 1960-1968 | 0.21* (0.07, 0.29) |
|  | Trend 2 | 1968-1980 | 0.52* (0.46, 0.73) |
|  | Trend 3 | 1980-1992 | 0.36* (0.30, 0.45) |
|  | Trend 4 | 1992-2014 | 0.26* (0.23, 0.28) |
|  | Trend 5 | 2014-2023 | 0.13 (-0.002, 0.19) |
|  | **AAPC** | **1960-2023** | **0.31* (0.30, 0.31)** |
