# Supplementary material for: A study of trends and projection of life expectancy and its association with socio-demographic index: Results from GBD study 2023
Source: PLoS One. 2026 Jun 3;21(6):e0347865. doi: 10.1371/journal.pone.0347865 (PMC13232855; doi:10.1371/journal.pone.0347865)
Supplement: S3 Table — Results of the Joinpoint regression models for trend analysis of life expectancy at birth by region from 1960 to 2023. (DOCX) [file pone.0347865.s003.docx]

**S3 Table. Results of the Joinpoint regression models for trend analysis of life expectancy at birth by region from 1960 to 2023.**

| Region | Trend | Period | APC (95% CI) |
| --- | --- | --- | --- |
| AFE | Trend 1 | 1960-1981 | 0.57* (0.51, 0.62) |
|  | Trend 2 | 1981-1984 | -0.78 (-1.10, 0.64) |
|  | Trend 3 | 1984-1987 | 1.34 (-0.81, 1.72) |
|  | Trend 4 | 1987-1998 | 0.10 (-0/27, 1.33) |
|  | Trend 5 | 1998-2004 | 0.89* (0.09, 1.21) |
|  | Trend 6 | 2004-2013 | 1.32* (0.44, 1.81) |
|  | Trend 7 | 2013-2023 | 0.61* (0.45, 0.87) |
|  | **AAPC** | **1960-2023** | **0.60* (0.59, 0.62)** |
| AFW | Trend 1 | 1960-1964 | 1.01* (0.89, 1.25) |
|  | Trend 2 | 1964-1967 | 0.26* (0.13, 0.54) |
|  | Trend 3 | 1967-1979 | 1.33* (1.28, 1.38) |
|  | Trend 4 | 1979-1983 | 0.67* (0.41, 1.06) |
|  | Trend 5 | 1983-1998 | 0.24* (0.19. 0.30) |
|  | Trend 6 | 1998-2010 | 0.86* (0.26, 0.91) |
|  | Trend 7 | 2010-2021 | 0.47* (0.32, 0.86) |
|  | Trend 8 | 2021-2023 | 0.99* (0.50, 1.21) |
|  | **AAPC** | **1960-2023** | **0.70* (0.69, 0.71)** |
| CEB | Trend 1 | 1960-1966 | 0.43* (0.29, 0.64) |
|  | Trend 2 | 1966-1994 | 0.07* (0.05, 0.09) |
|  | Trend 3 | 1994-2018 | 0.37* (0.35, 0.40) |
|  | Trend 4 | 2018-2021 | -1.09* (-1.31, -0.66) |
|  | Trend 5 | 2021-2023 | 1.92* (1.40, 2.38) |
|  | **AAPC** | **1960-2023** | **0.22* (0.21, 0.24)** |
| EAP | Trend 1 | 1960-1962 | 13.23*(12.87, 13.83) |
|  | Trend 2 | 1962-1980 | 1.12* (1.08, 1.15) |
|  | Trend 3 | 1980-1992 | 0.52* (0.49, 0.56) |
|  | Trend 4 | 1999-2015 | 0.37* (0.33, 0.42) |
|  | Trend 5 | 2015-2023 | 0.12 (-0.88, 0.21) |
|  | **AAPC** | **1960-2023** | **0.98* (0.97, 0.10)** |
| LCN | Trend 1 | 1960-1983 | 0.72* (0.70, 0.73) |
|  | Trend 2 | 1983-2004 | 0.51* (0.48, 0.53) |
|  | Trend 3 | 2004-2018 | 0.29* (0.25, 0.33) |
|  | Trend 4 | 2018-2021 | -1.37* (-1.55, -1.12) |
|  | Trend 5 | 2021-2023 | 2.48* (2.07, 2.86) |
|  | **AAPC** | **1960-2023** | **0.51* (0.50, 0.52)** |
| MEA | Trend 1 | 1960-1967 | 1.66* (1.46, 1.94) |
|  | Trend 2 | 1967-1987 | 1.15* (1.09, 1.20) |
|  | Trend 3 | 1987-2002 | 0.57* (0.50, 0.59) |
|  | Trend 4 | 2002-2018 | 0.37* (0.27, 0.59) |
|  | Trend 5 | 2018-2021 | -0.54 (-0.94, 0.59) |
|  | Trend 6 | 2021-2023 | 1.74* (0.18, 2.42) |
|  | **AAPC** | **1960-2023** | **0.80* (0.77, 0.82)** |
| NAC | Trend 1 | 1960-1969 | 0.07 (-0.03, 0.14) |
|  | Trend 2 | 1969-1978 | 0.47* (0.37, 0.65) |
|  | Trend 3 | 1978-2012 | 0.21* (0.20, 0.41) |
|  | Trend 4 | 2012-2018 | 0.04 (-0.11, 0.20) |
|  | Trend 5 | 2018-2021 | -0.92* (-1.12, -0.62) |
|  | Trend 6 | 2021-2023 | 1.14* (0.77, 1.49) |
|  | **AAPC** | **1960-2023** | **0.18* (0.18, 0.19)** |
| SAS | Trend 1 | 1960-1971 | 0.53* (0.13, 0.71) |
|  | Trend 2 | 1971-1982 | 1.21* (1.02, 2.16) |
|  | Trend 3 | 1982-2018 | 0.72* (0.69, 0.75) |
|  | Trend 4 | 2018-2021 | -1.10 (-1.71, 0.01) |
|  | Trend 5 | 2021-2023 | 2.82* (0.90, 4.06) |
|  | **AAPC** | **1960-2023** | **0.75* (0.70, 0.78)** |
