# Supplementary material for: A study of trends and projection of life expectancy and its association with socio-demographic index: Results from GBD study 2023
Source: PLoS One. 2026 Jun 3;21(6):e0347865. doi: 10.1371/journal.pone.0347865 (PMC13232855; doi:10.1371/journal.pone.0347865)
Supplement: S4 Table — Results of the Joinpoint regression models for trend analysis of life expectancy at birth by SDI from 1960 to 2023. (DOCX) [file pone.0347865.s004.docx]

**S4 Table. Results of the Joinpoint regression models for trend analysis of life expectancy at birth by SDI from 1960 to 2023.**

| SDI | Trend | Period | APC (95% CI) |
| --- | --- | --- | --- |
| Low SDI | Trend 1 | 1960-1983 | 0.52 (0.49, 0.54) |
|  | Trend 2 | 1983-1995 | 0.14 (0.07, 0.23) |
|  | Trend 3 | 1995-1998 | -0.59 (-0.77, -0.13) |
|  | Trend 4 | 1998-2018 | 0.54 (0.51, 0.58) |
|  | Trend 5 | 2018-2021 | -0.29 (-0.54, 0.12) |
|  | Trend 6 | 2021-2023 | 1.44 (0.84, 1.94) |
|  | **AAPC** | **1960-2023** | 0.39 (0.38, 0.40) |
| Low-middle SDI | Trend 1 | 1960-1974 | -0.14 (-0.19, -0.09) |
|  | Trend 2 | 1974-1982 | 0.29 (0.18, 0.58) |
|  | Trend 3 | 1982-1994 | -0.03 (-0.28, 0.03) |
|  | Trend 4 | 1994-2006 | 0.13 (0.08, 0.35) |
|  | Trend 5 | 2006-2021 | -0.23 (-0.28, -0.20) |
|  | Trend 6 | 2021-2023 | 1.32 (0.70, 1.68) |
|  | **AAPC** | **1960-2023** | 0.01 (-0.00, 0.02) |
| Middle SDI | Trend 1 | 1960-1981 | -0.20 (-0.26, -0.15) |
|  | Trend 2 | 1981-1988 | 0.53 (0.25, 1.25) |
|  | Trend 3 | 1988-1997 | -0.16 (-0.78, 0.04) |
|  | Trend 4 | 1997-2017 | 0.19 (0.10, 0.31) |
|  | Trend 5 | 2017-2021 | -1.09 (-1.76, 0.17) |
|  | Trend 6 | 2021-2023 | 1.12 (-0.45, 1.98) |
|  | **AAPC** | **1960-2023** | -0.00 (-0.03, 0.01) |
| High-middle SDI | Trend 1 | 1960-1986 | 0.10 (0.06, 0.16) |
|  | Trend 2 | 1986-1990 | -0.73 (-1.23, -0.15) |
|  | Trend 3 | 1990-2023 | 0.15 (0.12, 0.20) |
|  | **AAPC** | **1960-2023** | 0.07 (0.06, 0.09) |
| High SDI | Trend 1 | 1960-1970 | 0.32 (0.25, 0.40) |
|  | Trend 2 | 1970-1973 | -0.43 (-0.57, 0.01) |
|  | Trend 3 | 1973-1990 | 0.30 (0.25, 0.36) |
|  | Trend 4 | 1990-1999 | 0.05 (-0.22, 0.29) |
|  | Trend 5 | 1999-2018 | 0.17 (0.14, 0.30) |
|  | Trend 6 | 2018-2021 | -0.65 (-0.89, -0.29) |
|  | Trend 7 | 2021-2023 | 0.85 (0.30, 1.29) |
|  | **AAPC** | **1960-2023** | 0.17 (0.15, 0.18) |
