# Supplementary material for: A study of trends and projection of life expectancy and its association with socio-demographic index: Results from GBD study 2023
Source: PLoS One. 2026 Jun 3;21(6):e0347865. doi: 10.1371/journal.pone.0347865 (PMC13232855; doi:10.1371/journal.pone.0347865)
Supplement: S5 Table — Results of the Joinpoint regression models for trend analysis of life expectancy at birth by income group from 1960 to 2023. (DOCX) [file pone.0347865.s005.docx]

**S5 Table. Results of the Joinpoint regression models for trend analysis of life expectancy at birth by income group from 1960 to 2023.**

| Income | Trend | Period | APC (95% CI) |
| --- | --- | --- | --- |
| Low income | Trend 1 | 1960-1979 | 0.83 (0.80, 0.87) |
|  | Trend 2 | 1979-1984 | 0.06 (-0.40, 0.28) |
|  | Trend 3 | 1984-1989 | 0.87 (0.64, 1.30) |
|  | Trend 4 | 1989-1998 | 0.36 (0.07, 0.46) |
|  | Trend 5 | 1998-2007 | 1.22 (1.11, 1.39) |
|  | Trend 6 | 2007-2023 | 0.53 (0.48, 0.57) |
|  | **AAPC** | **1960-2023** | **0.68 (0.67, 0.69)** |
| Low-middle income | Trend 1 | 1960-1971 | 0.62 (0.36, 0.74) |
|  | Trend 2 | 1971-1982 | 1.12 (0.99, 1.60) |
|  | Trend 3 | 1982-2018 | 0.59 (0.57, 0.61) |
|  | Trend 4 | 2018-2021 | -0.74 (-1.15, -0.07) |
|  | Trend 5 | 2021-2023 | 2.10 (0.91, 2.92) |
|  | **AAPC** | **1960-2023** | **0.67 (0.64, 0.69)** |
| Middle income | Trend 1 | 1960-1962 | 7.16 (6.80, 7.63) |
|  | Trend 2 | 1962-1983 | 0.98 (0.96, 1.01) |
|  | Trend 3 | 1983-2018 | 0.48 (0.47, 0.49) |
|  | Trend 4 | 2018-2021 | -0.68 (-0.91, -0.27) |
|  | Trend 5 | 2021-2023 | 1.50 (0.93, 1.99) |
|  | **AAPC** | **1960-2023** | **0.83 (0.81, 0.84)** |
| High-middle income | Trend 1 | 1960-1962 | 12.11 (11.74, 12.65) |
|  | Trend 2 | 1962-1977 | 1.13 (1.09, 1.18) |
|  | Trend 3 | 1977-1985 | 0.74 (0.63, 1.03) |
|  | Trend 4 | 1985-2000 | 0.50 (0.45, 0.65) |
|  | Trend 5 | 2000-2018 | 0.39 (0.36, 0.44) |
|  | Trend 6 | 2018-2021 | -0.48 (-0.72, -0.05) |
|  | Trend 7 | 2021-2023 | 0.94 (0.20, 1.38) |
|  | **AAPC** | **1960-2023** | **0.96 (0.95, 0.98)** |
| High income | Trend 1 | 1960-1989 | 0.29 (0.28, 0.31) |
|  | Trend 2 | 1989-1994 | 0.05 (-0.12, 0.33) |
|  | Trend 3 | 1994-2013 | 0.32 (0.01, 0.44) |
|  | Trend 4 | 2013-2018 | 0.20 (0.09, 0.32) |
|  | Trend 5 | 2018-2021 | -0.52 (-0.67, -0.32) |
|  | Trend 6 | 2021-2023 | 0.78 (0.52, 1.03) |
|  | **AAPC** | **1960-2023** | **0.25 (0.25, 0.26)** |
