# Supplementary material for: A study of trends and projection of life expectancy and its association with socio-demographic index: Results from GBD study 2023
Source: PLoS One. 2026 Jun 3;21(6):e0347865. doi: 10.1371/journal.pone.0347865 (PMC13232855; doi:10.1371/journal.pone.0347865)
Supplement: S6 Table — Year-wise correlation between the Socio-Demographic Index and life expectancy at birth from 1960 to 2023. (DOCX) [file pone.0347865.s006.docx]

**S6 Table. Year-wise correlation between the Socio-Demographic Index and life expectancy at birth from 1960 to 2023.**

| **Year** | **Correlation (95% CI)** | **Year** | **Correlation (95% CI)** | **Year** | **Correlation (95% CI)** |
| --- | --- | --- | --- | --- | --- |
| 1960 | 0.89 (0.86, 0.92) | 1985 | 0.88 (0.85, 0.91) | 2010 | 0.86 (0.81, 0.89) |
| 1961 | 0.90 (0.87, 0.92) | 1986 | 0.89 (0.86, 0.92) | 2011 | 0.86 (0.82, 0.89) |
| 1962 | 0.90 (0.86, 0.92) | 1987 | 0.88 (0.84, 0.91) | 2012 | 0.87 (0.83, 0.90) |
| 1963 | 0.90 (0.87, 0.92) | 1988 | 0.86 (0.82, 0.89) | 2013 | 0.87 (0.83, 0.90) |
| 1964 | 0.90 (0.87, 0.93) | 1989 | 0.89 (0.86, 0.92) | 2014 | 0.86 (0.82, 0.89) |
| 1965 | 0.90 (0.87, 0.92) | 1990 | 0.89 (0.85, 0.91) | 2015 | 0.86 (0.82, 0.89) |
| 1966 | 0.90 (0.86, 0.92) | 1991 | 0.89 (0.85, 0.91) | 2016 | 0.86 (0.82, 0.89) |
| 1967 | 0.90 (0.86, 0.92) | 1992 | 0.87 (0.83, 0.90) | 2017 | 0.86 (0.81, 0.89) |
| 1968 | 0.89 (0.86, 0.92) | 1993 | 0.86 (0.82, 0.89) | 2018 | 0.88 (0.84, 0.90) |
| 1969 | 0.89 (0.85, 0.91) | 1994 | 0.85 (0.81, 0.89) | 2019 | 0.86 (0.82, 0.89) |
| 1970 | 0.88 (0.85, 0.91) | 1995 | 0.87 (0.84, 0.90) | 2020 | 0.87 (0.84, 0.90) |
| 1971 | 0.89 (0.85, 0.92) | 1996 | 0.87 (0.84, 0.90) | 2021 | 0.85 (0.80, 0.88) |
| 1972 | 0.89 (0.85, 0.91) | 1997 | 0.87 (0.83, 0.90) | 2022 | 0.82 (0.77, 0.86) |
| 1973 | 0.89 (0.86, 0.92) | 1998 | 0.86 (0.81, 0.89) | 2023 | 0.86 (0.82, 0.90) |
| 1974 | 0.89 (0.86, 0.92) | 1999 | 0.87 (0.83, 0.90) |  |  |
| 1975 | 0.86 (0.82, 0.89) | 2000 | 0.87 (0.83, 0.90) |  |  |
| 1976 | 0.84 (0.79, 0.88) | 2001 | 0.86 (0.82, 0.90) |  |  |
| 1977 | 0.85 (0.81, 0.89) | 2002 | 0.86 (0.82, 0.89) |  |  |
| 1978 | 0.85 (0.81, 0.89) | 2003 | 0.85 (0.81, 0.89) |  |  |
| 1979 | 0.88 (0.85, 0.91) | 2004 | 0.85 (0.81, 0.89) |  |  |
| 1980 | 0.89 (0.85, 0.91) | 2005 | 0.85 (0.81, 0.89) |  |  |
| 1981 | 0.89 (0.86, 0.92) | 2006 | 0.85 (0.81, 0.88) |  |  |
| 1982 | 0.88 (0.85, 0.91) | 2007 | 0.85 (0.81, 0.89) |  |  |
| 1983 | 0.89 (0.85, 0.91) | 2008 | 0.85 (0.81, 0.89) |  |  |
| 1984 | 0.88 (0.84, 0.91) | 2009 | 0.83 (0.78, 0.87) |  |  |
